# Case of scombroid syndrome mimicking an anaphylactic fish allergy

**DOI:** 10.1186/s12245-025-00930-3

**Published:** 2025-07-03

**Authors:** Véronique Gingras, Louis Marois

**Affiliations:** 1 Division of Emergency Medicine, Faculty of Medicine, Université Laval, Québec, Canada; 2 Division of Specialized Medicine, Faculty of Medicine, Université Laval, Québec, Canada; 3 Allergy and Immunology Department, CHU de Québec, H1340-2705 boulevard Laurier, Québec, Canada; 4 CHU de Québec Research Center, Université Laval, Québec, Canada

**Keywords:** Scombroid syndrome, Fish allergy, Anaphylaxis, Food allergy

## Abstract

**Background:**

Scombroid syndrome, a pseudo-allergic reaction caused by histamine-contaminated fish, is often mistaken for type I hypersensitivity reactions due to overlapping clinical presentations. Unlike IgE-mediated anaphylaxis, Scombroid syndrome results from histamine production by bacterial histidine decarboxylase in improperly preserved fish. It accounts for 5% of U.S. food poisoning cases and manifests within 30 min to two hours post-ingestion.

**Case presentation:**

Symptoms include rash, pruritus, vomiting, and tongue swelling, as exemplified by a case of a 30-year-old woman misdiagnosed with anaphylaxis after consuming salmon. However, typically mild and self-limiting, severe cases may involve bronchospasm and arrhythmias. Diagnosis hinges on patient history, symptom onset, and absence of food allergy markers, such as elevated tryptase. Management includes antihistamines and supportive care; epinephrine and corticosteroids are reserved for severe reactions.

**Conclusion:**

Clinicians must distinguish Scombroid syndrome from anaphylaxis to ensure accurate treatment, emphasizing proper fish preservation to prevent outbreaks.

## Background

Hypersensitivity reactions are exaggerated immune responses to antigens. Type I hypersensitivity reactions constitute a significant proportion of hypersensitivity reactions and are the most observed in emergency departments. They are characterized as acute IgE-mediated reactions that occur when antigens bind to specific IgEs present on the surfaces of mast cells and basophils. These reactions can lead to life-threatening conditions, including anaphylactic shock, angioedema, and airway obstruction [1]. Other reactions that can mimic type I hypersensitivity include mast cell-independent allergic reactions resulting from the ingestion of exogenous histamine produced outside the human body, known as Scombroid syndrome [2].

The scombroid syndrome is a pseudo-allergic poisoning caused by the consumption of fish contaminated with histamine. Histamine is not typically found in fish; rather, it is produced by histidine decarboxylase, an enzyme derived from bacteria in the fish’s gills and gastrointestinal tract. Histidine decarboxylase (HDC) exhibits enzymatic activity under conditions favorable to bacterial proliferation, particularly at storage temperatures ranging from 20 °C to 30 °C. In such environments, histamine-producing bacteria, including *Photobacterium damselae*, can generate toxic concentrations of histamine within 2 to 4 h. Importantly, histamine is thermostable and resistant to conventional food processing methods such as cooking, freezing, or canning. Consequently, stringent temperature control below 4 °C is essential throughout the supply chain to mitigate the risk of scombroid poisoning [3]. This type of poisoning accounts for approximately 5% of all reported food poisoning cases in the United States and around 40% of poisonings resulting from fish consumption. Scombroid poisoning is triggered by the ingestion of poorly preserved fish from the *Scombroidae* and *Scomberesocidae* families, including tuna, bonito, mackerel, albacore, skipjack, herring, sardines, anchovies, bluefish, sea urchins, and mahi-mahi. Salmon may also be implicated, though more rarely [4]. The true incidence of Scombroid syndrome is believed to be higher than reported, as many cases go unrecorded due to the transient nature of the symptoms. Although the clinical course of Scombroid syndrome is typically self-limiting and benign, vascular compromise, bronchospasm, and arrhythmias have been documented, underscoring the importance of accurately distinguishing these conditions. In particular, the presence of atopic comorbidities such as asthma may potentially exacerbate the severity of symptoms [5].

## Case presentation

A 30-year-old woman presented to the emergency room in the evening with facial and thoracic rash, pruritus, profuse vomiting, difficulty breathing, and swelling of the tongue. She reported having eaten salmon salad from a takeout restaurant two hours prior, a meal she had consumed in the past many times. She has no known allergies, asthma or eczema. She began vomiting and experiencing swelling of her tongue 30 min after her meal. Her vital signs were BP 159/80, HR 162, SpO2 99% without the use of supplemental oxygen, Temperature 37.5 °C. She receives an initial dose of 0.3 mg epinephrine IM twice within 6 min, along with 50 + 25 mg Benadryl IV, 50 mg ranitidine IV, and 125 mg methylprednisolone IV. Epinephrine was administered despite the absence of hemodynamic instability or evident airway compromise, in the context of suspected anaphylaxis. Although the patient did not experience desaturation, nebulized epinephrine was administered for dyspnea and decreased breath sounds. She remained hypertensive and tachycardic, showing sinus tachycardia on the EKG (114–168 bpm). She received a total of four doses of epinephrine IM as she reported worsening symptoms, particularly swelling and itching of the tongue, shortly after each dose. As part of the initial emergency management for suspected anaphylaxis, and in light of the patient’s profuse vomiting, a total of 3 L of 0.9% sodium chloride was administered. A rhinoscopy did not reveal any airway edema. An epinephrine infusion of 0.05 mcg/kg/min was initiated and tapered over an hour. She was also given 200 ml of 50% dextrose in 0.9% saline, 50 mg of prednisone orally for the morning, 50 mg of ranitidine IV every 8 h, and 50 mg of diphenhydramine orally every 8 h. Laboratory results showed slight metabolic acidosis with an increased anion gap, which resolved by the following morning. The patient was discharged the next day with an adrenaline auto-injector, a referral to the allergology clinic, and advised to avoid consuming salmon until her appointment. All skin prick tests for fish allergens, performed during the allergology consultation three months later, were negative. Additionally, specific IgE testing for salmon yielded a result below the detection threshold (< 0.35 kU/L), supporting the diagnosis of scombroid syndrome, a non-IgE-mediated reaction. The patient subsequently reintroduced salmon into her diet without incident, further ruling out an IgE-mediated fish allergy.

## Discussion and conclusion

This case reminds us that food reactions can be of various pathophysiological mechanisms and illustrates elements contributing to the differential diagnosis of IgE-mediated anaphylaxis (Table 1). However, very few cases are reported in the context of emergency medicine evaluation [6, 7]. In particular, this case highlights the frequent diagnostic confusion between scombroid syndrome and food-induced anaphylaxis, as well as the importance of recognizing subtle clinical nuances to guide more appropriate patient management. The patient reported consuming salmon at least once weekly for several years. Anaphylactic reactions to regularly consumed foods are considered uncommon, as repeated exposure to dietary antigens is typically associated with the development of oral tolerance rather than sensitization [8]. Nonetheless, fish allergy remains one of the most prevalent food allergies among adults in North America [9]. Besides, complete avoidance of all fish prior to allergological evaluation would have been more appropriate then salmon only. There were no other allergens present in her meal. Additionally, she noted feeling itching 30 min after her first bite. While this is not an absolute rule, Scombroid syndrome symptoms typically appear within 30 min to two hours, while fish allergies initiate symptoms within just a few minutes. Moreover, hypotension is rare in Scombroid syndrome, and despite experiencing recurrent symptoms, this patient was never hypotensive. However, the tryptase level was not tested in the emergency department. It could have assisted clinicians in differentiating Scombroid syndrome from anaphylaxis, although a negative tryptase assay result does not exclude an IgE-dependent allergy. An anaphylactic reaction mediated by IgE causes an increase in the tryptase value in the minutes and for a few hours following the onset of symptoms, unlike Scombroid syndrome, which does not depend on a release of molecules by the patient’s mast cells but well from an exogenous supply of ingested histamine. This difference argues in favor of systematically testing tryptase when a patient presents with symptoms of acute food reactions. Although their administration is often necessary in the face of an acute food reaction, corticosteroids and epinephrine are not strictly indicated for mild cases that are preferably treated using antihistamines, both H1 and H2. For moderate to severe cases, intravenous administration of antihistamines (H1 and H2) is advised, along with isotonic crystalloid fluids, if the patient is hypotensive. In cases presenting with respiratory edema, hypotension, and bronchospasm, epinephrine (0.3 mg IM for adults; 0.01 mg/kg IM for children) and corticosteroids (60 mg prednisone orally for adults or 125 mg IV methylprednisolone sodium succinate) should be administered. In the event of bronchospasm, beta agonists (e.g., salbutamol) may be used via inhalation [1]. Experience suggests that signs and symptoms of poisoning typically resolve within 30 min of antihistamine administration in these patients. Though specific evidence is lacking, administering oral antihistamines (e.g., loratadine or cetirizine) for one to two days is a reasonable approach to prevent the recurrence of symptoms caused by ongoing toxin absorption from the gastrointestinal tract.

**Table 1:** Scombroid syndrome and fish anaphylaxis: similarities and differences

Characteristics	Scombroid syndrome	Anaphylaxis
Onset	30 min to 2 h	Within few minutes
Patients	Everyone who eats the contaminated fish	Only patients sensitized to fish
History of food allergy	No	Possible
Pathogenesis	Not immune-mediated	IgE-mediated
Mediator	Histamine	Histamine, tryptase, leukotrienes, bradykinins, cytokines…
Rash	Usually mild: facial and upper trunk flushing	Generalized: Pruritus, urticarial, angioedema
Hypotension	Uncommon	Frequent
Laboratory	High plasma levels of histamine	High serum levels of tryptase
Treatment	Antihistamines + supportive therapy, if necessary	IM adrenaline + supportive therapy, if needed
Prevention measures	Optimal management of fish	Avoidance of the allergen, Epipen

Scombroid syndrome and anaphylactic reactions can be confusing due to their initial presentations in the emergency department. However, their pathogenic mechanisms and treatments differ. Several key differences can assist clinicians in the emergency department in distinguishing between the two conditions:

1. The onset of symptoms is generally faster in fish allergies.
2. Different individuals may experience similar symptoms after consuming fish in Scombroid syndrome.
3. Patients typically have no history of food allergies in Scombroid syndrome.
4. Symptoms are usually mild to moderate in Scombroid syndrome.
5. Hypotension is uncommon in Scombroid syndrome.
6. Tryptase serum levels are negative in Scombroid syndrome.

Correctly establishing the diagnosis is crucial for effective patient management. Scombroid poisoning is generally a benign, self-limiting condition; however, due to reports of distributive shock, bronchospasm, and cardiac arrhythmias, early diagnosis and treatment are essential.

## Data Availability

No datasets were generated or analysed during the current study.

## Abbreviations

BP: Blood pressure
EKG: Electrocardiogram
HR: Heart rate
IgE: Immunoglobulin E
IM: Intramuscular
IV: Intravenous
SpO2: Oxygen saturation
